# Development and validation of a radiomic model for the diagnosis of dopaminergic denervation on [18F]FDOPA PET/CT

**DOI:** 10.1007/s00259-022-05816-7

**Published:** 2022-05-14

**Authors:** Victor Comte, Hugo Schmutz, David Chardin, Fanny Orlhac, Jacques Darcourt, Olivier Humbert

**Affiliations:** 1 Department of Nuclear Medicine, Centre Antoine Lacassagne, Université Côte d’Azur, Nice, France; 2 Laboratoire TIRO UMR E4320, Université Côte d’Azur, Nice, France; 3 Laboratoire d’Imagerie Translationnelle en Oncologie (LITO) U1288, Institut Curie, Inserm, Université Paris-Saclay, Orsay, France

**Keywords:** 18FDOPA, Parkinsonian syndromes, Machine learning, Radiomics, Texture analysis

## Abstract

**Purpose:**

FDOPA PET shows good performance for the diagnosis of striatal dopaminergic denervation, making it a valuable tool for the differential diagnosis of Parkinsonism. Textural features are image biomarkers that could potentially improve the early diagnosis and monitoring of neurodegenerative parkinsonian syndromes. We explored the performances of textural features for binary classification of FDOPA scans.

**Methods:**

We used two FDOPA PET datasets: 443 scans for feature selection, and 100 scans from a different PET/CT system for model testing. Scans were labelled according to expert interpretation (dopaminergic denervation versus no dopaminergic denervation). We built LASSO logistic regression models using 43 biomarkers including 32 textural features. Clinical data were also collected using a shortened UPDRS scale.

**Results:**

The model built from the clinical data alone had a mean area under the receiver operating characteristics (AUROC) of 63.91. Conventional imaging features reached a maximum score of 93.47 but the addition of textural features significantly improved the AUROC to 95.73 (*p* < 0.001), and 96.10 (*p* < 0.001) when limiting the model to the top three features: GLCM_Correlation, Skewness and Compacity. Testing the model on the external dataset yielded an AUROC of 96.00, with 95% sensitivity and 97% specificity. GLCM_Correlation was one of the most independent features on correlation analysis, and systematically had the heaviest weight in the classification model.

**Conclusion:**

A simple model with three radiomic features can identify pathologic FDOPA PET scans with excellent sensitivity and specificity. Textural features show promise for the diagnosis of parkinsonian syndromes.

**Supplementary Information:**

The online version contains supplementary material available at 10.1007/s00259-022-05816-7.

## Introduction

Parkinson’s disease (PD) is the second most common neurodegenerative disease worldwide with a rising prevalence due in part to population ageing [1]. In addition to the well-known cardinal motor symptoms, it is associated with numerous non-motor symptoms such as autonomic dysfunction, cognitive impairment and sleep disorders [2], and is responsible for increasing disability and mortality [3]. The main pathological feature of PD is dopaminergic cell loss in the substantia nigra pars compacta that leads to dopamine deficiency in the basal ganglia; this process is exponential and starts years before the clinical diagnosis, as motor symptoms only appear after the loss of 50% of nigral dopamine neurons [4]. The development of biomarkers is considered critical to diagnose patients at an early stage, to predict the rapidity of neuron loss and to monitor the progression of the disease; all of which are essential for the development of disease-modifying drugs [5, 6].

6-[^18^F]FDOPA is a PET radiotracer that reflects vesicular dopamine storage within the striatum [7] and has been shown to correlate with the severity of PD symptoms [8]. For the diagnosis of dopaminergic denervation, it has been estimated to reach 95% sensitivity [9]. Visual analysis relies for the most part on assessing the homogeneity of striatal uptake and the point of maximum uptake [10]. However, it is intrinsically limited by the fact that the striatum is both the pathological target and the reference area for the internal uptake level. Semi-quantification using striatal-to-occipital ratios (SOR) helps in assessing the global and local uptake level, but the heterogeneity of uptake within the striatum remains difficult to gauge.

The computation of statistical textural features is a way to quantify heterogeneity in images, including 3D PET data [11]. Briefly, the process involves the calculation of a matrix that captures the relationships between two or more voxels, in different directions; then, various image biomarkers are computed directly from this matrix. The high-throughput extraction of a large number of these quantitative biomarkers, or features, defines the field of radiomics [12]. Depending on the disease, imaging technique, acquisition protocol and numerous other factors, different features may be useful, such that machine learning algorithms are often applied to identify one or more relevant features [13]. Texture analysis has shown promising results for DAT SPECT [14–16], and has been applied to FDOPA PET for glioma analysis [17] but has not yet been applied to FDOPA PET for striatal study.

The aim of this study was to identify textural features that could function as biomarkers of dopaminergic denervation on FDOPA PET, and to measure their performances.

## Material and methods

### Patients

Two datasets were retrospectively analysed, an exploration dataset and a testing dataset. The exploration dataset consisted of all patients consecutively referred for striatal FDOPA PET/CT to the nuclear medicine department of the Centre Antoine Lacassagne, Nice, France, from January 8, 2020, to April 14, 2021. Inclusion stopped after reaching 450 subjects. We excluded six patients with an abnormal acquisition time, as it was shown to affect textural features [18, 19]. We also excluded one patient who had poor scan quality (visually unreadable). The final number of patients in the exploration dataset was 443, with 171 scans (39%) interpreted as positive and 272 scans (61%) interpreted as negative.

Clinical data were collected from patients on the day of their referral, using a shortened version of the Movement Disorder Society–sponsored revision of the Unified Parkinson’s Disease Rating Scale (MDS-UPDRS) [20] (detailed in the electronic supplementary material). Twenty-five motor and non-motor symptoms were quantified between 0 and 4, for a maximum total score of 100.

The second dataset was reserved for testing the model and comprised 100 patients referred to the same centre between May 10, 2019, and August 28, 2019, with scans acquired on a different PET/CT system (see below). Patients were selected in a consecutive manner, and the same exclusion criteria were applied. After reaching 60 negative scans, only positive scans were included in order to reproduce the positive/negative ratio of the exploration dataset. No clinical data were collected in this group.

### Image acquisition

For the exploration dataset, FDOPA PET/CT images were acquired on a Biograph Vision 600 (Siemens, Erlangen, Germany). The images were obtained after a period of 85–95 min following the intravenous injection of 3 MBq/kg of 3,4-dihydroxy-6-[^18^F]fluoro-L-phenylalanine (DOPAVIEW® AAA company). PET acquisition time was 6 min. The acquisition matrix size was 512 × 512. Images were reconstructed using OSEM, with 12 iterations and 5 subsets, and a Gaussian filter with FWHM = 3 mm was applied. Time-of-flight (TOF) correction was applied, but not point spread function (PSF) correction. The reconstructed voxel size was 0.709 × 0.709 × 2.00 mm.

For the test dataset, FDOPA PET/CT images were acquired on a Biograph mCT 40 (Siemens, Erlangen, Germany). The images were obtained after a period of 85–95 min following the intravenous injection of 2 MBq/kg of 3,4-dihydroxy-6-[^18^F]fluoro-L-phenylalanine (DOPAVIEW® AAA company). PET acquisition time was 10 min. The acquisition matrix size was 512 × 512. Images were reconstructed using OSEM, with 6 iterations and 24 subsets, and a Gaussian filter with FWHM = 4 mm was applied. No TOF or PSF corrections were applied. The reconstructed voxel size was 0.795 × 0.795 × 2.03 mm.

In both cases, protein-containing foods were banned 4 h prior to the procedure, as per EANM guidelines. Patients with no contraindications received 100 mg of carbidopa 1 h before injection [21]; the number of patients who did not receive carbidopa was 22 in the exploration dataset (5%) and 4 in the test dataset (4%).

For all scans, ground truth was the interpretation of a nuclear medicine specialist with extensive experience in FDOPA imaging (J.D.). This expert had access to clinical data, MRI data when available and semi-quantification software based on automatic positioning of atlas-derived striatal VOI and comparison of the SOR to a reference database (Siemens Scenium Striatal analysis). For this study, subjects with evidence of uni- or bilateral dopaminergic denervation were considered positive, and those with normal scans or evidence of vascular parkinsonism were considered negative.

### Image processing

VOI segmentation and feature extraction was performed on LIFEx version 6.3 [22]. On each side, a threshold of 40% of the SUVmax was applied over the caudate and putamen. When this led to the creation of several distinct VOI, only the largest VOI was kept. All left-side and right-side features were later averaged for each subject. Forty-three features were computed for each 3D VOI, including shape descriptors (*n* = 5), first-order histogram statistics (*n* = 7) and second- and higher-order statistics from the grey-level co-occurrence matrix (GLCM) (*n* = 6), grey-level run-length matrix (GLRLM) (*n* = 11), neighbourhood grey-level difference matrix (NGLDM) (*n* = 3) and grey-level zone-length matrix (GLZLM) (*n* = 11). They are listed, along with their Image Biomarker Standardisation Initiative (IBSI) denomination [23], in the electronic supplementary material.

To assess texture robustness, several computation settings were tried. All histogram features were computed after absolute discretisation [24, 25] between a lower bound of 0 and an upper bound of 10, using either 32, 64 or 128 grey levels (GLs) [26]. Voxel size was either reconstructed to be isotropic (1 × 1 × 1 mm or 2 × 2 × 2 mm) [27], or left with default values. For the features derived from the grey-level co-occurrence matrix (GLCM), three different distances were tried: 1, 2 and 5 [14]. Thus, in total, the 43 features were computed 21 times for all 443 patients in the exploration dataset (Table 1).

**Table 1:** List of the combinations of pre-processing parameters that were studied. *GLCM* grey-level co-occurrence matrix

		32 grey levels	64 grey levels	128 grey levels
GLCM distance = 1	Default voxel size	32–1-default	64–1-default	128–1-default
	1 × 1 × 1 mm	32–1-111	64–1-111	128–1-111
	2 × 2 × 2 mm	32–1-222	64–1-222	128–1-222
GLCM distance = 2	1 × 1 × 1 mm	32–2-111	64–2-111	128–2-111
	2 × 2 × 2 mm	32–2-222	64–2-222	128–2-222
GLCM distance = 5	1 × 1 × 1 mm	32–5-111	64–5-111	128–5-111
	2 × 2 × 2 mm	32–5-222	64–5-222	128–5-222

Independently for each dataset, features were standardised by removing the mean and scaling to unit variance.

### Data analysis

Statistical differences between groups were tested with a *z* test for binary variables and, for continuous variables, with the Student *t*-test or the Mann–Whitney *U* test as appropriate. The correlations between features were computed with Pearson’s correlation coefficient, for each parameter set, and averaged for each feature, giving a 43 × 43 correlation matrix. Feature reproducibility across different parameter sets was assessed using the concordance correlation coefficient (CCC) [28]. This index measures the agreement between two methods of measuring the same continuous variable [29]. A CCC of 1 indicates perfect agreement; a value below 0.9 is generally considered poor [30].

In order to perform feature selection, we used logistic regression models with L1 penalisation, also known as LASSO [31]. The LASSO performs its own feature selection by setting the coefficients of less useful features to zero, which allows for good performance even against a large number of features [32] while ultimately yielding a simple and understandable model [33]. To optimise the model parameters, we used the SAGA solver, a popular variant of the stochastic average gradient solver adapted for the LASSO [34]. We systematically performed fivefold cross-validation and the regularisation strength was selected by the cross-validation for each iteration. Furthermore, we performed 1000 bootstrap resamples in order to estimate the confidence interval and the probability of variable selection [35].

After performing feature selection on the exploration dataset, the chosen features were tested on the test dataset, using the optimal parameter combination and a standard logistic regression model. We reported AUROC, sensitivity, specificity and balanced accuracy.

Descriptive statistics including the mean of features and the AUROC of individual features were only computed for one parameter set. This set was chosen according to the best performance reached by the models, using all features. This was also the reference set for the CCC.

For the clinical data only, we had to compensate for missing values using mean imputation.

All statistical tests and model building were performed on Python version 3.7.11 using free and open-source packages. The source code is available on: https://github.com/tirolab/FDOPA-PET-analysis.

## Results

The population is described in Table 2. For the exploration dataset, out of 443 patients, 171 (39%) showed unilaterally or bilaterally reduced uptake, while 272 (61%) had a normal FDOPA distribution. Overall, 56% were male, 95% were right-handed and 19% had diabetes. At the time of referral, 16% of patients were taking antiparkinsonian medication, and 8% were taking antipsychotic drugs. The median age was 74, with IQR 68–80. Five percent of patients could not receive carbidopa before injection. When comparing positive and negative patients, we found the sex ratio, as well as the proportion of patients with diabetes, and the proportion of patients taking antiparkinsonian drugs or antipsychotic drugs, to be significantly different.

**Table 2:** Characteristics of the population, with *p* values of the *z* test for binary variables and Mann–Whitney *U* test for age; in bold when significant for alpha = 0.05

	All scans	Positive scans	Negative scans	*p* value
Exploration dataset				
* n*	443	171	272	
Mean age	72.7	72.8	72.6	0.305
Males (%)	56	65	50	**0.002**
Right-handed (%)	95	97	94	0.254
Type 2 diabetes (%)	19	14	23	**0.05**
Taking antiparkinsonian drug (%)	16	26	10	** < 0.001**
Taking antipsychotic drug (%)	8	2	11	**0.001**
Received carbidopa (%)	95	97	94	0.292
Test dataset				
* n*	100	40	60	
Mean age	73.9	75.1	73.1	0.179
Males (%)	51	60	45	0.142
Received carbidopa (%)	96	98	95	0.532

In the test dataset, the proportion of positive and negative scans was identical to that of the exploration dataset, by design. The sex ratio, the mean age and the proportion of patients who received carbidopa were also similar to the exploration dataset. No data was collected on the presence of diabetes, right- or left-handedness or drugs taken.

The mean (SD) SUVmean across all VOIs was 2.61 (0.76) and mean (SD) SUVmax was 4.56 (1.25). Mean (SD) VOI volume was 12.9 mL (3.7).

**Fig. 1:**
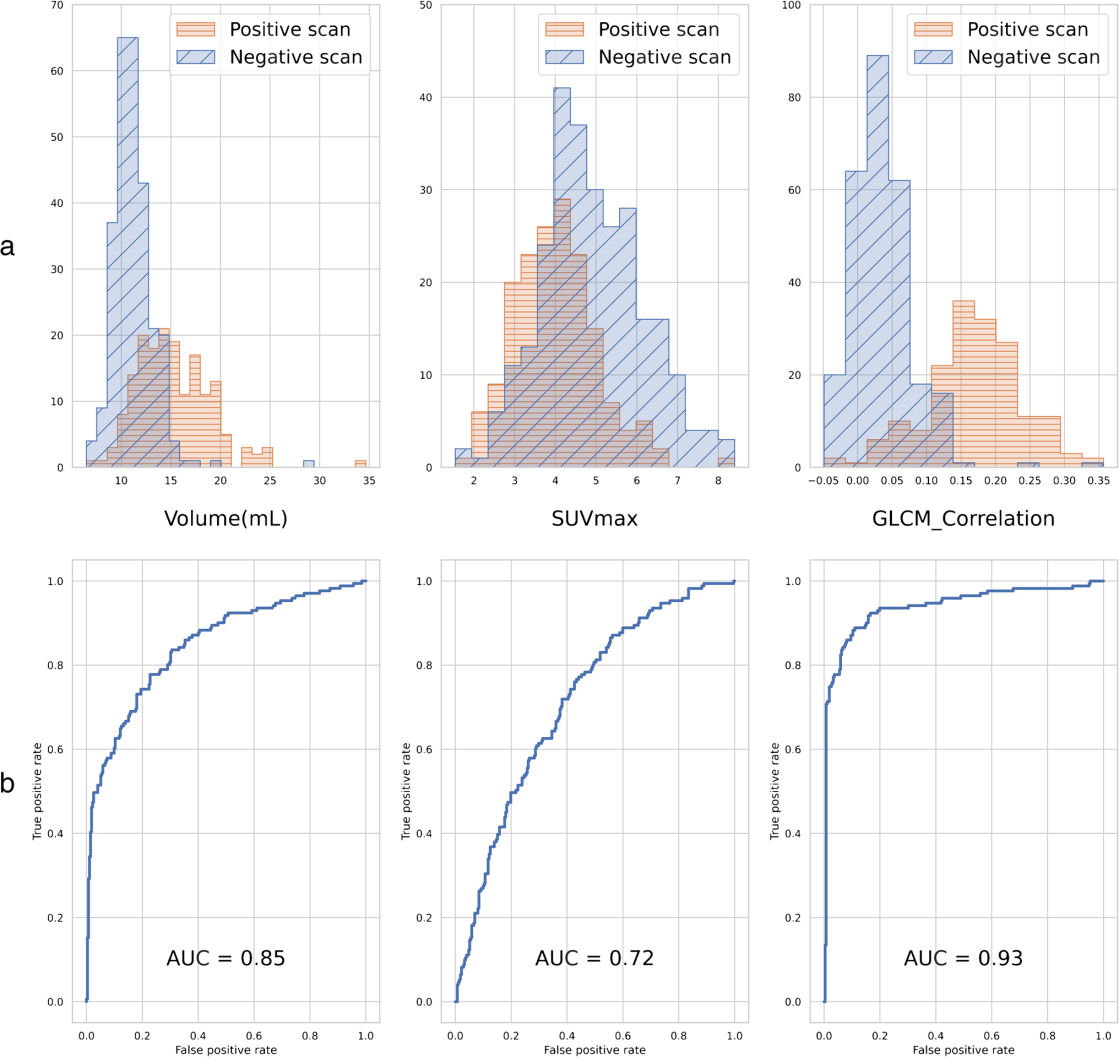
Histograms of Volume, SUVmax and GLCM_Correlation for positive and negative scans (**a**) and ROC curves with AUROC for the same variables (**b**)

The histograms of the conventional features showed considerable overlap between the “positive” and “negative” populations; some are shown in Fig. 1a. ROC curves were drawn to estimate the predictive value of features in isolation (Fig. 1b). The features with the highest AUC were GLCM_Correlation (93.91), Skewness (93.22) and Sphericity (91.91).

The mean clinical score was 22 for patients with negative scans and 20 for patients with positive scans. The difference between the mean values was not significant (Student’s *p*-value: 0.18). The mean (SD) AUC reached by the clinical model was 63.91 (5.27).

**Fig. 2:**
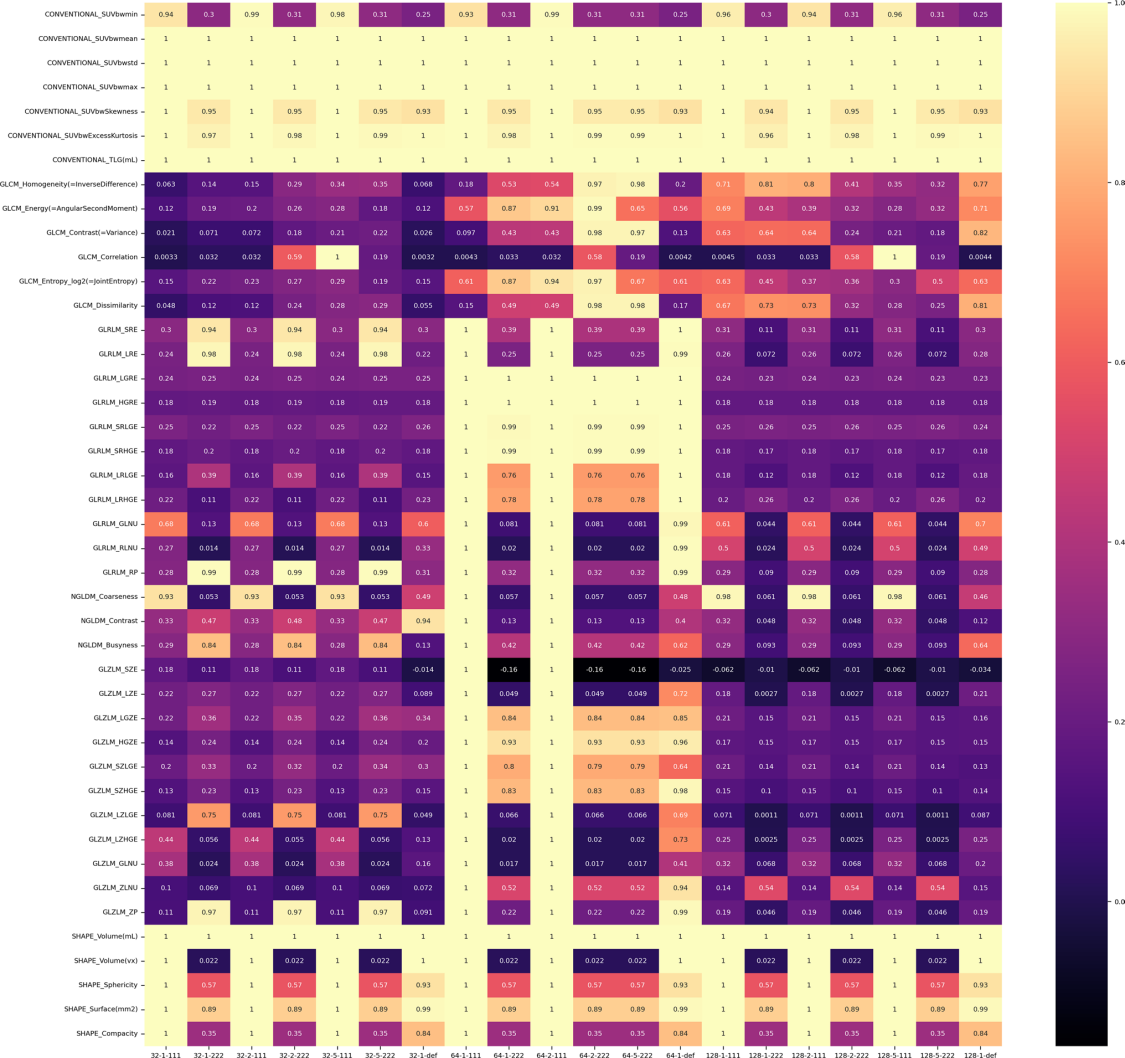
Concordance correlation coefficient for the 64–5-111 set versus all other sets, for all 43 features

Concerning the impact of the pre-processing of the image data, the CCC results are shown in Fig. 2. First-order histogram features (except for SUVmin) were very robust, but overall textural features were highly affected by the choice of pre-processing parameters, with most features showing CCCs below 0.9, indicating poor agreement.

A “conventional” series of models were trained to study the performance of non-textural features, as a baseline performance. They used the following features: SUVmin, SUVmean, SUVmax, Total Volume Uptake (TVU) (the product of the SUVmean by the volume in millilitres, named Total Lesion Glycolysis in LifeX), Volume (mL), Surface, Sphericity and Compacity. Twenty-one models were trained, one for every pre-processing parameter set. The highest score was reached with the 128–2-222 set, with a mean (SD) AUROC of 93.47 (2.17) (Table 3).

**Table 3:** Mean and standard deviation of AUC scores for models 1 and 2 according to parameter set. Highest score in bold

	Conventional		All features	
	Mean AUROC	SD	Mean AUROC	SD
32–1-111	92.45	2.37	94.16	2.04
32–1-222	93.36	2.16	94.90	1.86
32–2-111	92.50	2.47	94.92	1.90
32–2-222	93.25	2.13	95.01	1.57
32–5-111	92.44	2.35	95.22	1.64
32–5-222	93.32	2.27	95.50	2.00
32–1-default	92.95	2.28	94.04	2.14
64–1-111	92.41	2.43	94.26	1.68
64–1-222	93.24	2.15	94.72	1.90
64–2-111	92.52	2.34	94.92	1.79
64–2-222	93.36	2.21	94.99	2.02
64–5-111	92.60	2.37	**95.73**	1.93
64–5-222	93.23	2.17	95.65	1.88
64–1-default	92.77	2.45	94.26	2.09
128–1-111	92.63	2.42	94.60	1.66
128–1-222	93.24	2.17	94.71	1.73
128–2-111	92.50	2.39	94.89	1.54
128–2-222	**93.47**	2.17	94.44	2.05
128–5-111	92.55	2.37	95.17	2.14
128–5-222	93.28	2.16	94.96	1.88
128–1-default	92.96	2.28	94.50	1.57

A second series of models including all features was trained following the same training process as the first series. The highest score was reached with the 64–5-111 set, with a mean (SD) AUROC of 95.73 (1.93) (Table 3). There was a significant difference between this score and that of the best-performing “conventional” model (*p* < 0.001). Excluding the 22 patients who had not received carbidopa did not significantly change the results. For this set, Table 4 shows the first five features ordered by the probability of their coefficient being non-zero, as well as the average coefficient of each feature when non-zero. The top feature was GLCM_Correlation, which was selected in every instance, and had the highest average coefficient. This feature was selected 100% of the time in all parameter sets (except the ones with a default (non-isotropic) voxel size), giving it the highest average probability of selection. The complete list of selected features is available as electronic supplementary material.

**Table 4:** Top five features for the 64–5-111 set ordered by probability of inclusion, and average coefficient when selected. *TVU* total volume uptake, the product of the SUVmean by the volume in millilitres, equivalent to total lesion glycolysis

Feature	Probability	Coefficient
GLCM_Correlation	1.00	2.47
CONVENTIONAL_SUVbwSkewness	0.98	0.66
SHAPE_Compacity	0.97	− 0.67
NGLDM_Contrast	0.84	− 0.51
TVU (mL)	0.58	− 1.17

**Fig. 3:**
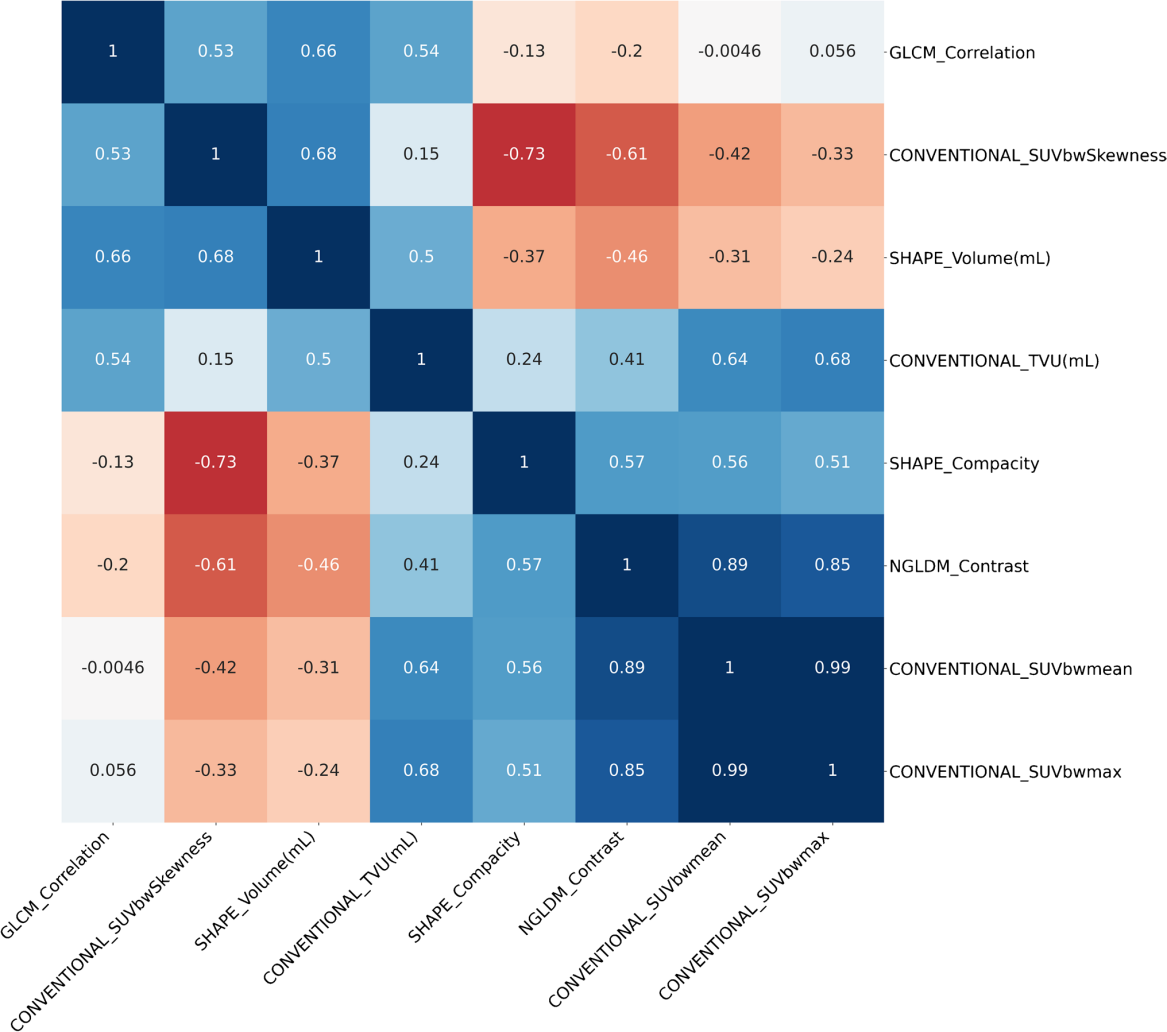
Pearson’s correlation coefficients between the top five features, as well as SUVmean, SUVmax and Volume, averaged across all 21 parameter sets. Positive coefficients are in blue, negative coefficients in red. Clustering follows the unweighted pair group method with arithmetic mean

The Pearson correlations between the top five features, as well as SUVmean, SUVmax and Volume, are shown in Fig. 3. The full figure with all features is available as electronic supplementary material.

We then built a “simplified” model, using only the top three features, which had a probability of being included close to 100%. Those features were GLCM_Correlation, Skewness and Compacity (Table 4). This model was only trained on the 64–5-111 set as this was the pre-processing parameter combination that enabled the best performance in the previous model. The mean (SD) AUC score was 96.06 (1.79) using cross-validation on the exploration dataset. There was a significant difference between this score and that of the best-performing “conventional” model (*p* < 0.001).

Finally, our “simplified” model was trained on the entire exploration dataset and tested on the testing dataset. The AUROC was 98.21 with a balanced accuracy of 95.83%, a sensitivity of 95.00% and a specificity of 96.67%.

## Discussion

In this study, we explored the feasibility and the value of a radiomic approach for the diagnosis and quantification of dopaminergic denervation.

The clinical scores revealed that the patients of our population had moderate symptoms of parkinsonism at the time of referral. The clinical score was not significantly different between patients with positive and negative scans, and the model built with the clinical features had a mediocre AUC score. This can be explained by the fact that most patients are referred for FDOPA imaging when the clinical presentation is atypical. Likewise, the fact that there were significantly more patients taking antiparkinsonian medication in the positive group reflects the real-life situation of neurologists using L-DOPA therapy as a diagnostic test. The proportion of patients taking antipsychotic drugs was higher in the negative group because those patients are more likely to present with drug-induced parkinsonism. The higher proportion of male subjects in the positive group is in agreement with the 3:2 sex ratio described in the literature [2].

The histograms and ROC curves showed the sub-optimal performance of Volume and SUVmax, considered independently, to classify the patients. The higher volume in positive patients is explained by the segmentation method: a fixed relative threshold will yield a greater volume when the SUV values decrease.

We proved that the addition of textural features to our model significantly improved its performance. This enhanced performance did not change when we restricted the entry variables to the top three features identified by the LASSO.

GLCM_Correlation was the only feature to be systematically selected and systematically had the heaviest weight in the model. For a given grey-level co-occurrence matrix, the equation for Correlation along one direction is:

$${Correlation}_{GLCM}=\sum_{i}\sum_{j}\frac{(i-{\mu }_{i})\bullet (j-{\mu }_{j})\bullet GLCM(i,j)}{{{\sigma }_{i}\bullet \sigma }_{j}}$$

with *μ* and *σ* respectively the mean and standard deviation for row i or column j.

This feature reflects the linear dependency of grey levels within the VOI: in theory, its value is closer to 0 when the spatial distribution of values in the VOI is random. In our study, positive scans had on average a higher GLCM_Correlation value. One hypothesis is that the uptake gradient observed in PD [10] could be responsible for the increase in Correlation along the antero-posterior axis. However, in 3D, the features are averaged over 13 directions, making this relationship less obvious.

We showed that this feature is only moderately correlated with morphological features, as the highest coefficient was 0.66, for the correlation with Volume (Fig. 3). Thus, GLCM_Correlation is not simply a proxy of the VOI volume—a well-known pitfall in radiomics [36]—nor other conventional indices. GLCM_Correlation has previously been described as a robust and independent feature in other clinical scenarios [37–40]: our results are in agreement with those findings, despite the relatively small size of VOI in this study, which has been raised as a potential limit of textural features [24]. Pre-processing settings had a high impact on the feature values, as shown by CCC, but this is not a surprising result as other authors have previously demonstrated the inevitable effect that the number of grey levels and the voxel size have on textural values [18, 41]. However, this did not affect the feature selection and model scores. This may mean that the change in absolute values did not significantly alter the relative order of features [42]. It is important to note that non-isotropic voxel sizes were the only parameter change that resulted in GLCM_Correlation not being selected 100% of the time.

Finally, we tested our results on an external dataset with the same positive/negative ratio, as per expert recommendations [33, 43]. With 95.00% sensitivity with 96.67% specificity, we showed that our model still performs well on scans acquired with a different PET/CT system and a different protocol, as long as features are normalised beforehand.

To our knowledge, this is the first radiomics study to explore the diagnosis of dopaminergic denervation on FDOPA PET. For DAT SPECT, Rahmim et al. [15, 16] showed the potential of radiomics for the prediction of the UPDRS score; they also found Correlation to be a feature of interest, but only for the MOCA score. Before them, Martinez-Murcia et al. [14] had published promising results comparing several feature selection methods, but did not identify Correlation as relevant for distinguishing positive from negative DaTSCANs.

Our study has several strengths. The cohort design ensured that our dataset was close to the target population, with patients scanned at an early stage and an authentic ratio of positive and negative scans. We included a large number of patients, complying with the expert recommendation of including more than ten patients for each feature [13]. Our classification followed the visual analysis of an expert helped by the most recent semi-quantitative analysis software. The simplicity of the 40% isocontour method is likely to ensure a good interobserver reproducibility, although we did not test it. We studied feature correlation and robustness, and assessed their usefulness using a series of simple but powerful models. Lastly, we tested our model on scans acquired on a different PET/CT system with different parameters, which is an essential part of the radiomics process [43, 44]. To account for the differences of the two systems, we scaled features independently for the exploration and testing dataset. The similarity of our results on the two sets is in favour of the good generalisability of the model. It should be noted, however, that in order to reproduce our results in a different centre, a minimum of 30 patients will be necessary to ascertain the mean and variance of each feature in the new centre, in order to apply standardisation on the features before using the model.

We should also point out some limits to this study. Firstly, we chose to average the features computed independently on both striatal regions. Thus, the difference between patients with strongly asymmetric dopaminergic denervation and healthy patients was less noticeable than if we had chosen the most pathological side for each patient: this could have reduced our power in this study. Secondly, we did not study the impact of different acquisition parameters, image reconstruction settings and segmentation methods on model performance, all of which have been found to influence textural features [45, 46]. Finally, the clinical impact of our model remains to be tested.

## Conclusion

Using a LASSO approach, we were able to train a model based on only three conventional and textural features, which could predict dopaminergic denervation as visually assessed by a medical expert, with a mean AUROC of 96.06. Testing the model on an independent dataset yielded high performances, with an AUROC of 96.00, a sensitivity of 95.00% and a specificity of 96.67%. Combining textural and conventional features significantly improved the model compared to using conventional features alone. The most important textural feature for the models was GLCM_Correlation which we found to be independent and robust. Further research is needed to confirm the clinical usefulness of our model for the diagnosis of dopaminergic denervation on FDOPA PET scans.

## Supplementary Information

Below is the link to the electronic supplementary material.

Supplementary file1 (DOCX 838 KB)

## Data Availability

The datasets generated during this study are available from the corresponding author on reasonable request.

## Code availability

https://github.com/tirolab/FDOPA-PET-analysis
