# Supplementary material for: Development and validation of a radiomic model for the diagnosis of dopaminergic denervation on [18F]FDOPA PET/CT
Source: Eur J Nucl Med Mol Imaging. 2022 May 14;49(11):3787–96. doi: 10.1007/s00259-022-05816-7 (PMC9399031; doi:10.1007/s00259-022-05816-7)

# Clinical questionnaire

Our clinical questionnaire included the following questions taken from the official French translation of the Movement Disorder Society-sponsored revision of the Unified Parkinson's Disease Rating Scale (MDS-UPDRS) [1].

*Symptoms assessed by the investigator (12 questions)*

Part IA

1.1 Cognitive impairment

1.2 Hallucinations and psychosis

1.3 Depressed mood

1.4 Anxious mood

1.5 Apathy

Part III

3.1 Speech

3.3 Rigidity

3.5 Hand movements

3.9 Arising from chair

3.10 Gait

3.13 Posture

3.18 Constancy of rest tremor

*Self-administered questionnaire (10 questions)*

Part IB

1.8 Daytime sleepiness

1.9 Pain and other sensations

1.11 Constipation problems

1.13 Fatigue

Part II

2.1 Speech

2.3 Chewing and swallowing

2.7 Handwriting

2.10 Tremor

2.11 Getting out of bed, a car, or a deep chair

2.12 Walking and balance

1. Goetz CG, Tilley BC, Shaftman SR, Stebbins GT, Fahn S, Martinez-Martin P, et al. Movement Disorder Society-sponsored revision of the Unified Parkinson’s Disease Rating Scale (MDS-UPDRS): scale presentation and clinimetric testing results. Mov Disord. 2008;23:2129–70.

# Features included in this study

| **Name in LifeX** | **IBSI nomenclature** |
| --- | --- |
| **Shape Features** | **Morphological features** |
| SHAPE_Volume(mL) | Volume |
| SHAPE_Volume(vx) | Volume (voxel counting) |
| SHAPE_Sphericity | Sphericity |
| SHAPE_Surface(mm2) | Surface area |
| SHAPE_Compacity | N/A |
| **Conventional Indices** | **Intensity-based statistical features** |
| CONVENTIONAL_SUVbwmin | Minimum intensity |
| CONVENTIONAL_SUVbwmean | Mean intensity |
| CONVENTIONAL_SUVbwstd | N/A |
| CONVENTIONAL_SUVbwmax | Maximum intensity |
| CONVENTIONAL_SUVbwSkewness | Intensity skewness |
| CONVENTIONAL_SUVbwExcessKurtosis | Excess intensity kurtosis |
| CONVENTIONAL_TLG(mL) | N/A |
| **Grey Level Co-occurrence Matrix** | **Grey level co-occurrence based features; 3D-avg** |
| GLCM_Homogeneity(=InverseDifference) | Inverse difference |
| GLCM_Energy(=AngularSecondMoment) | Angular second moment |
| GLCM_Contrast(=Variance) | Contrast |
| GLCM_Correlation | Correlation |
| GLCM_Entropy_log2(=JointEntropy) | Joint entropy |
| GLCM_Dissimilarity | Dissimilarity |
| **Grey Level Run Length Matrix** | **Grey level run length based features; 3D-avg** |
| GLRLM_SRE | Short runs emphasis |
| GLRLM_LRE | Long runs emphasis |
| GLRLM_LGRE | Low grey level run emphasis |
| GLRLM_HGRE | High grey level run emphasis |
| GLRLM_SRLGE | Short run low grey level emphasis |
| GLRLM_SRHGE | Short run high grey level emphasis |
| GLRLM_LRLGE | Long run low grey level emphasis |
| GLRLM_LRHGE | Long run high grey level emphasis |
| GLRLM_GLNU | Grey level non-uniformity |
| GLRLM_RLNU | Run length non-uniformity |
| GLRLM_RP | Run percentage |
| **Neighbourhood Grey Level Difference Matrix** | **Neighbourhood grey tone difference based features; 3D** |
| NGLDM_Coarseness | Coarseness |
| NGLDM_Contrast | Contrast |
| NGLDM_Busyness | Busyness |
| **Grey Level Zone Length Matrix** | **Grey level size zone based features; 3D** |
| GLZLM_SZE | Small zone emphasis |
| GLZLM_LZE | Large zone emphasis |
| GLZLM_LGZE | Low grey level zone emphasis |
| GLZLM_HGZE | High grey level zone emphasis |
| GLZLM_SZLGE | Small zone low grey level emphasis |
| GLZLM_SZHGE | Small zone high grey level emphasis |
| GLZLM_LZLGE | Large zone low grey level emphasis |
| GLZLM_LZHGE | Large zone high grey level emphasis |
| GLZLM_GLNU | Grey level non-uniformity |
| GLZLM_ZLNU | Zone size non-uniformity |
| GLZLM_ZP | Zone percentage |

Features included in this study, with their LIFEx and Image Biomarker Standardisation Initiative (IBSI) denominations. Features noted as N/A are not defined in the IBSI reference manual. TLG = total lesion glycolysis (Volume(mL) multiplied by the mean intensity)

# Full list of features for the 64-5-111 set ordered by probability of inclusion, and average coefficient when selected

|  | **proba** | **score** |
| --- | --- | --- |
| **GLCM_Correlation** | 1 | 2.46821 |
| **CONVENTIONAL_SUVbwSkewness** | 0.98 | 0.658925 |
| **SHAPE_Compacity(onlyFor3DROI)** | 0.97 | -0.67112 |
| **NGLDM_Contrast** | 0.84 | -0.51017 |
| **CONVENTIONAL_TLG(mL)(onlyForPETorNM)** | 0.58 | -1.17433 |
| **GLZLM_GLNU** | 0.56 | 0.554924 |
| **GLZLM_SZLGE** | 0.52 | -0.51767 |
| **GLZLM_LGZE** | 0.51 | -0.33172 |
| **GLZLM_LZHGE** | 0.48 | -0.48952 |
| **CONVENTIONAL_SUVbwmin** | 0.43 | 0.143017 |
| **GLZLM_SZE** | 0.42 | -0.17501 |
| **GLRLM_RLNU** | 0.41 | -0.33876 |
| **CONVENTIONAL_SUVbwExcessKurtosis** | 0.39 | -0.0381 |
| **NGLDM_Busyness** | 0.38 | -0.55391 |
| **SHAPE_Sphericity(onlyFor3DROI))** | 0.36 | -0.45462 |
| **NGLDM_Coarseness** | 0.36 | -0.32607 |
| **CONVENTIONAL_SUVbwmax** | 0.32 | 0.616741 |
| **GLZLM_SZHGE** | 0.24 | 0.422057 |
| **GLCM_Dissimilarity** | 0.23 | 0.664586 |
| **GLZLM_LZLGE** | 0.22 | 0.517697 |
| **SHAPE_Volume(vx)** | 0.21 | -0.17548 |
| **SHAPE_Volume(mL)** | 0.21 | -0.17548 |
| **GLZLM_ZLNU** | 0.18 | -0.31195 |
| **GLZLM_ZP** | 0.18 | -0.34905 |
| **GLZLM_HGZE** | 0.16 | 0.32513 |
| **GLCM_Contrast(=Variance)** | 0.15 | 0.360058 |
| **GLRLM_LRLGE** | 0.14 | 0.382135 |
| **GLRLM_GLNU** | 0.14 | 0.258646 |
| **GLCM_Energy(=AngularSecondMoment)** | 0.14 | 0.662347 |
| **GLRLM_LRHGE** | 0.13 | -0.51621 |
| **CONVENTIONAL_SUVbwstd** | 0.11 | -0.29361 |
| **GLCM_Entropy_log2(=JointEntropy)** | 0.11 | 0.194517 |
| **GLCM_Homogeneity(=InverseDifference)** | 0.1 | -0.51072 |
| **GLRLM_HGRE** | 0.1 | -0.19651 |
| **GLRLM_LRE** | 0.1 | 0.354139 |
| **GLRLM_SRHGE** | 0.1 | -0.12034 |
| **GLZLM_LZE** | 0.09 | 0.148936 |
| **GLRLM_SRLGE** | 0.08 | -0.12471 |
| **GLRLM_RP** | 0.08 | 0.006544 |
| **GLRLM_SRE** | 0.06 | -0.23908 |
| **CONVENTIONAL_SUVbwmean** | 0.06 | 0.085177 |
| **GLRLM_LGRE** | 0.03 | 0.023489 |
| **SHAPE_Surface(mm2)(onlyFor3DROI)** | 0.03 | 0.199113 |

# Complete list of Pearson correlations between features


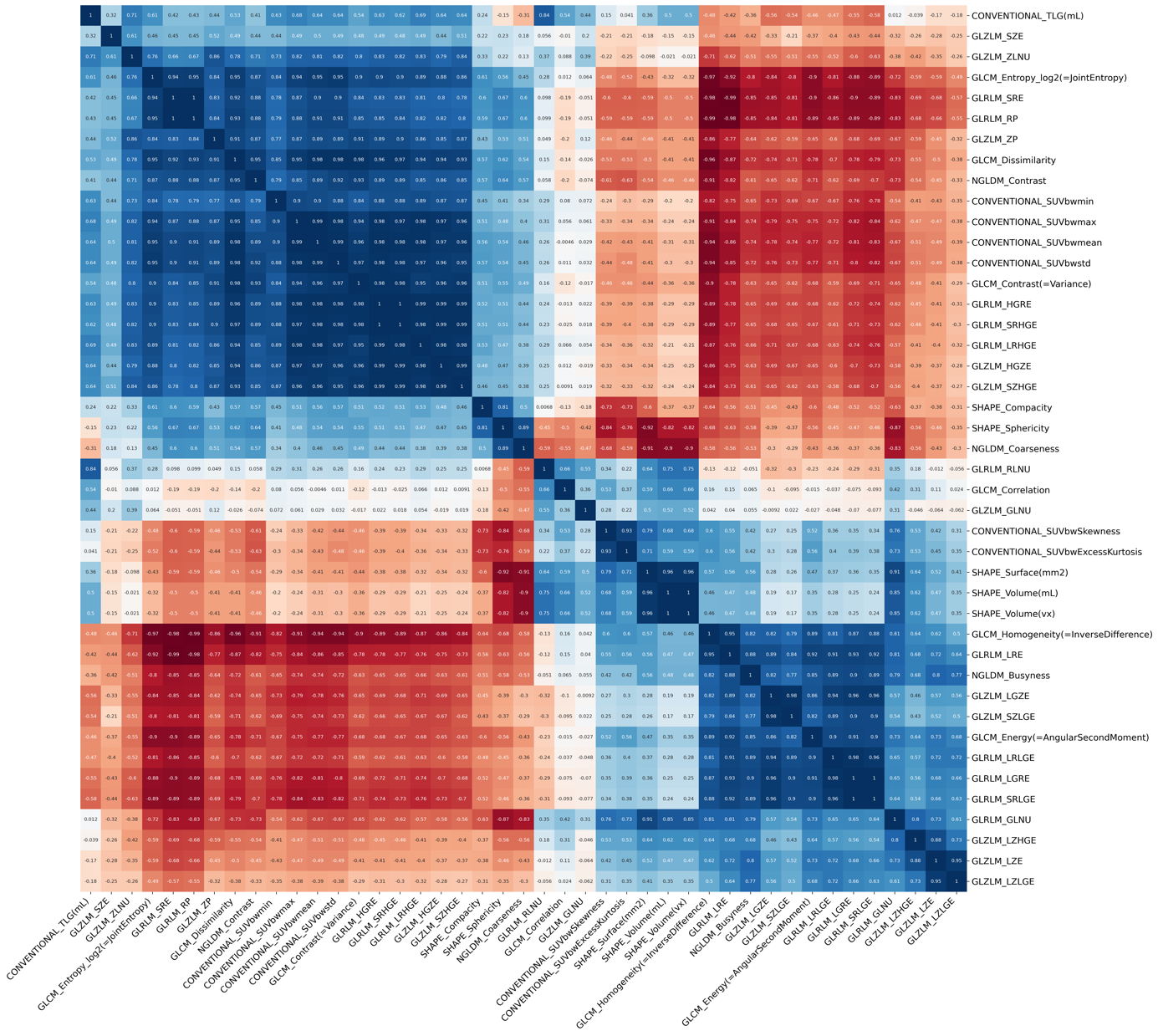

Supplement: Supplementary file 1 — Supplementary file1 (DOCX 838 KB) [file 259_2022_5816_MOESM1_ESM.docx]
